# The Availability and Quality of Food Labelling Components in the Canadian E-Grocery Retail Environment

**DOI:** 10.3390/nu13082611

**Published:** 2021-07-29

**Authors:** Jennifer J. Lee, Mavra Ahmed, Tianyi Zhang, Madyson V. Weippert, Alyssa Schermel, Mary R. L’Abbé

**Affiliations:** 1 Department of Nutritional Sciences, Temerty Faculty of Medicine, University of Toronto, Toronto, ON M5S 1A1, Canada; jennjm.lee@mail.utoronto.ca (J.J.L.); mavra.ahmed@utoronto.ca (M.A.); madyson.weippert@mail.utoronto.ca (M.V.W.); a.schermel@gmail.com (A.S.); 2 Joannah & Brian Lawson Centre for Child Nutrition, University of Toronto, Toronto, ON M5S 1A1, Canada; 3 Dalla Lana School of Public Health, University of Toronto, Toronto, ON M5S 1A1, Canada; tianyimph.zhang@mail.utoronto.ca

**Keywords:** online grocery, food labelling, e-commerce, e-grocery, food retail environment

## Abstract

Background: Although packaged foods sold in retail stores must follow food labelling regulations, there are no e-grocery food labelling regulations to mandate and standardize the availability and presentation of product information. Therefore, the objective of the study was to evaluate the availability and quality of food labelling components in the Canadian e-grocery retail environment. Methods: A sample of fresh and pre-packaged products was identified on eight leading grocery retail websites in Canada, to assess the availability and quality of food labelling components. Results: Out of 555 product searches, all products were accompanied by product images with front-of-pack images more readily available (96.0%) than back-of-pack (12.4%) and other side panel images (3.1%). The following mandatory nutrition information was available for 61.1% of the products: nutrition facts table (68.8%), ingredient (73.9%), and allergen (53.8%) information. The majority of the nutrition information was available after scrolling down, clicking additionally on the description page, or viewing only as an image. Date markings were not available; packaging material information was available for 2.0% of the products. Conclusions: There was wide variability and inconsistencies in the presentation of food labelling components in the e-grocery retail environment, which can be barriers in enabling Canadians to make informed purchasing decisions.

## 1. Introduction

With rapid expansions and the use of e-commerce during the COVID-19 pandemic [1], many grocery retailers have expanded their traditional brick-and-mortar retail stores with online platforms. E-grocery shopping can be a convenient shopping method, particularly for those with time constraints [2] or those concerned about minimizing human exposure during a pandemic [1]. However, there is limited information on the e-grocery retail environment, defined as the online sale of grocery products.

Food labelling, which provides information about the characteristics of the food, is the main method of communication between manufacturers and consumers [3], and is regarded as an important tool in facilitating healthy food choices by consumers [4,5]. As part of the Codex Alimentarius Commission to protect the health of consumers and to promote fair practices in the food trade, the Codex Committee on Food Labelling (CCFL) was established to provide regularly updated international practice and guidelines on labelling of foods to minimize false, misleading, or deceptive information presented to consumers [6,7]. The CCFL provides guidelines on various aspects of labelling, including the type (e.g., name of foods, ingredients, date marking, and storage information), content (e.g., claims, advertisements) and the presentation (e.g., legibility, location) of information [7]. The CCFL Guidelines are widely adopted across the world by over 180 countries to set country-specific labelling regulations [7,8]. Detailed guidance on the components of food labelling has been published by the Food and Agriculture Organization of the United Nations (FAO) in the book, “*Handbook on Food Labelling to Protect Consumers*” [9]; however, none of the components apply to foods sold on e-commerce websites (although an appeal at the CCFL 45th and 46th sessions began working on this topic [10,11]). In line with the CCFL Guidelines, Canadian food labelling regulations, including *Food and Drug Regulations (FDR)* [12] and *Safe Food for Canadians Regulations (SFCR)* [13], mandate standardized food labelling practices to ensure accurate, consistent, and transparent product information related to food character, value, composition, and safety are clearly communicated to Canadians. Although the current guidelines and regulations do not outline specific instructions for the e-grocery retail environment, per se, the scope of the labelling regulations should be similarly extended to the e-grocery retail environment to protect and inform consumers. However, the labelling conditions for foods and beverages in the e-grocery retail environment are unknown.

Comprehensive product information on the e-grocery retail environment is particularly important, as logistical and technical limitations of online shopping can restrict consumers’ choices. Contrary to brick-and-mortar stores, consumers cannot physically evaluate products while grocery shopping online, which tends to limit purchases of fresh produce and may drive more frequent purchases of pre-packaged foods and beverages [2,14,15]. E-grocery shoppers have shown to be less price-sensitive, more brand loyal, and less likely to explore new foods than brick-and-mortar shoppers [15], demonstrating potential barriers in promoting healthy purchasing behaviors among e-grocery shoppers. The behavioral differences between e-grocery and brick-and-mortar shoppers may, in part, be related to the lack of or difficulties in accessing product information on retail websites. In the U.S., the mandatory nutrition facts label was only present for 85% of the products on 12 grocery websites, with most of the nutrition facts labels available after scrolling or with an additional click on the product description page [16]. With limited studies examining the availability of food labelling components in the e-grocery retail environment, the objective of this study was to examine the availability and quality of food labelling components of products sold by leading Canadian grocery websites.

## 2. Materials and Methods

### 2.1. Study Design

A cross-sectional analysis of the availability and quality of food labelling components of foods and beverages sold on leading Canadian grocery websites was conducted. Seven retailers were included in the study: four leading Canadian grocery retailers, as shown through 2019 total company sales, and three retailers with leading online food sales in 2018, collectively representing over 80% of the grocery market share in Canada [17]. One of the retailers (Costco^®^, Costco Wholesale Canada Ltd., Nepean, ON, Canada) offered two levels of services: standard website offering limited services (i.e., no fresh produce available and a minimum 2-day delivery option); and the extended services (i.e., offering wider availability of products with fresh produce and a 1-day delivery option) in partnership with Instacart Inc. Therefore, eight websites (i.e., standard Costco^®^; Costco^®^ powered by Instacart; Grocery Gateway by Longo’s, Longo’s Brothers Fruit Markets Inc., Vaughan, ON, Canada; Loblaws^®^, Loblaws Companies Ltd., Brampton, ON, Canada; Metro, Metro Inc., Montreal, QB, Canada; No Frills^®^, Loblaws Companies Ltd., Brampton, ON, Canada; Voilà by Sobeys, Empire Company Ltd., Stellarton, NS, Canada; and Walmart, Walmart Canada Corp., Mississauga, ON, Canada) from seven retailers were selected for review. Two research assistants searched for the selected products on eight websites, with the location set for downtown Toronto, ON, Canada between 18 and 21 December 2020.

### 2.2. Data Collection

A pre-defined sample of 85 fresh and pre-packaged foods, composed of 2–5 foods from each of the 23 categories according to Health Canada’s Table of References Amount categories [18], were selected to assess the availability and quality of food labelling components on grocery websites. Of 85 selected products, 63 brand-specific products of category market leaders and 22 generic or private-label products without any specific brand were selected. Generic products included products with no brands (e.g., fresh produce) and private retail grocery-brands (e.g., President’s Choice^®^ for Loblaws Inc., Great Value^TM^ for Walmart Inc.). Table S1 shows the list of the pre-selected 85 products examined in the study.

### 2.3. Outcome Measures

The eight selected websites were examined for general accessibility (e.g., membership status, language), delivery and/or pick-up information (e.g., availability, fee), the general presentation of groceries (e.g., grocery categories), and other available consumer-friendly features (e.g., order history, save lists, substitution availability, consumer ratings or feedback). Privacy and security information was assessed for the accessibility of privacy policy, data gathering and sharing practices, and data security based on the privacy best practices guide by the Online Trust Alliance [19].

The total number and the availability of label images were tabulated, including front-of-pack, back-of-pack, and side panels. The quality of product images was assessed based on the legibility, categorized as follows: (1) can read everything on images without zooming in; (2) can only read everything on images after zooming in; and (3) cannot read everything even after zooming in. Key labelling information required on food labels, as per the *FDR* [12], were examined to compare the availability of information: nutrition facts table (NFt), ingredient information, allergen information, nutrition and health claims, date marking, storage information, and package information (including recycling information). NFt, ingredient, and allergen statements were counted for availability, then further assessed for quality based on the following 4 categories, as previously reported [16]: (1) located on product information page with no scrolling required; (2) located on the product information page, but scrolling is required to view on the same page; (3) information is available within one click away from the product information page; and (4) only available as part of the product image. Nutrition, health, and other food claims, regulated by Health Canada or Agriculture and Agri-Food Canada (e.g., “no added sugars”, “high source of fiber”, “organic”), were assessed for the presence as part of the website product description (i.e., not part of the food images). The availability of product recommendations made by retailers, including similar, complimentary, or other non-related sponsored products, was examined. Date markings were examined for any of the following terms as indicated by the *FDR* [12]: “best before”, “expired by”, “sell by”, “prepared on”, “freeze by”, and “manufactured on.” The availability of storage information (e.g., “keep refrigerated”, “shelf-stable”) was assessed for all products and for products requiring refrigeration. The availability of package information (e.g., glass, plastic, cardboard) and recycling information were assessed. The availability of price information was examined for cost per product and per reference amount (e.g., per 100 g, 100 mL, 1 lb).

### 2.4. Statistical Analysis

General quality of the websites and the availability of product information were presented as the proportion for all products and by brand type. Considering the role that manufacturers and retailers may have in displaying food labelling information to consumers, brand types (i.e., brand-specific vs. generic and private-label products) and websites were treated as independent variables for additional analysis of the examined outcomes. The effect of brand types on the outcomes was assessed by comparing brand-specific vs. generic and private-label products using Student’s t-test for continuous variables and Fisher’s exact test for categorical variables. The effect of websites on the outcomes was assessed by comparing the outcomes of brand-specific products across 8 websites, using one-way analysis of variance for continuous variables and chi-squared tests for categorical variables. All analyses were conducted using RStudio (version 1.4.1106, RStudio, Boston, MA, USA). Statistical significance was defined at *p* < 0.05.

## 3. Results

### 3.1. General Website Quality

Characteristics for the eight websites are shown in Table 1 (detailed characteristics by website are shown in Table S2). Although all eight websites were available in English, only five of those websites were available in French (one of two official languages in Canada). All eight websites required a member profile and two required a membership fee ($60–$120/y). Pick-up and delivery services were available on five and seven websites, respectively, with some requiring minimum purchasing amounts ($30–$50 and $35–$50, respectively) and/or service fees ($2.97–$5.00 and $3.00–$15.50, respectively) depending on the date, time, and location of the delivery.

Several consumer-friendly features were available. Order history and ‘save for later’ options were available on seven websites, allowing consumers to easily re-order previous purchases, keep track of frequently consumed foods, and mark foods for later purchases. Six websites offered a substitution function, where retailers would identify alternatives if the foods ordered were not available. Two websites allowed consumers to leave feedback and/or ratings using a five star system; however, it was not clear whether the feedback was strictly related to the quality or characteristics of the product or the website. Among an average of 17.5 (SD: 4.9) food categories per website, Fruits and Vegetables, Prepared Foods, and Meat and Seafood were commonly found within the top three food categories in all websites.

A detailed privacy statement was available on all eight websites; however, only six websites offered a French version, and three websites offered a plain English summary version. All websites indicated the collection of “personally identifiable information” and sharing of users’ data with a third party. Only six websites disclosed data security details in the company’s privacy policy statement.

### 3.2. Overall Availability and Quality of Labelling Components

Out of 85 pre-selected products, a total of 555 products were searched on the 8 websites (413 brand-specific and 142 generic or private-label products). A summary of the availability and quality of product information is shown in Table 2 (detailed information by brand type and website are shown in Tables S3 and S4, respectively). All products were accompanied by product images (mean (SD): 2.3 (1.9)). Front-of-pack product images were available 96% of the time, while back-of-pack and other side panel images were not readily available (12.4% and 3.1%, respectively). The quality of most front-of-pack images was poor, with 81.1% of the products having images that could not be read even after zooming in.

Of 506 products required to declare nutrition information as per the *FDR* [3], all mandatory nutrition information was available 61.1% of the time. NFt was available 68.8% of the time, but needed to scroll-down (35.3%) or click a link (39.9%) from the information page; or was viewed as a product image (24.7%). Ingredient information was available 73.9% of the time without needing to scroll (0.2%), after scrolling down (22.7%), at least one click away (66.6%), or only as a product image (10.4%). Allergen information was available 53.8% of the time without scrolling (0.7%), after scrolling down (15.6%), at least one click away (65.2%), or only as a product image (19.3%). Nutrient, health, and other food claims regulated by Health Canada or Agriculture and Agri-Food Canada (e.g., “no added sugars”, “high source of fiber”, “organic”) were included in text form as part of the product description (45.0%), or represented with company-specific symbols (12.4%); we noted that non-regulated product characteristics were also available (e.g., “keto-friendly”, “low FODMAP diet”) and presented in a similar fashion as the regulated claims (i.e., in text or in symbols). Further, retailers’ recommendations were available in the form of “alternatives”, “similar products”, or “sponsored” 40.5% of the time. Date markings were not available on any of the products. Price per product was available for all products; however, price per reference amount or unit was present 67.4% of the time. Storage information was available for 21.8% of all products and available for 25.1% of the refrigerated products. Packaging and recycling information for packages were not readily available (2.0% and 1.8%, respectively).

### 3.3. Differences between Brand Types

A summary of the availability and quality of key labelling information categorized by brand-specific and generic or private-label product types are shown in Table S3. The total number of available product images was different between brand-specific and generic or private-label products (*p* < 0.001), where brand-specific products included more product images (mean (SD): 2.5 (2.1) vs. 1.6 (1.0)). Generic or private-label products offered more images that were legible without zoom features (14.1% vs. 1.9%; *p* < 0.001); however, the difference may have been related to the lack of small-print texts and/or symbols available in labels for fresh produce. Brand-specific foods more frequently showed front-of-pack and back-of-pack images than generic or private-label products (*p* ≤ 0.01).

The availability of ingredient information was significantly higher in brand-specific products than generic or private-label products (*p* < 0.01); however, all other availability and quality measures of mandatory nutrition information were not significant between the two groups (*p* ≥ 0.06). Nutrition and health claims using text were more frequently found with brand-specific foods (*p* = 0.002), whereas symbols were more frequently found with generic foods (*p* = 0.03). There was no difference in the availability of retailers’ product recommendations (*p* = 0.49). Price per reference amount was more frequently available for brand-specific products than generic or private-label products (*p* < 0.001). Overall storage information was less frequently available for brand-specific products (*p* < 0.001); however, storage information for refrigerated products was more frequently available for brand-specific products compared with generic or private-label products. Packaging and recycling information was rarely presented in either brand type of products (<2.4%; *p* ≥ 0.30).

### 3.4. Differences between Websites

The availability and quality of key labelling information across different websites was assessed by searching 63 unique brand-specific products on the 8 websites (413 product searches). Details of the availability and quality of food labelling components on the eight websites are shown in Table S4. The total number of available product images was different between websites, with an average of 1–4.7 images per product available (*p* < 0.001). There was no difference in the availability of front-of-pack images (*p* = 0.70); however, back-of-pack and side images were different among websites, as the images were occasionally available only on three websites (*p* < 0.001). Image quality, availability and quality of nutrition information, nutrition and health claims, price per reference amount, retailer recommendations, storage information, and packaging information were all different across websites (*p* < 0.001) with a wide variability, ranging from never offered (i.e., 0%) to frequently offered (i.e., >80%). Although the availability of recycling information was not different among websites (*p* = 0.45), the overall availability was very low, with a range of 0–3.2% across websites.

## 4. Discussion

The objective of the study was to assess the availability and quality of key labelling components in the Canadian e-grocery retail environment by searching 85 products on 8 websites of 7 grocery retailers in Canada, for a total of 555 product searches. Although many consumer-friendly features (e.g., delivery and re-ordering past orders) were available to improve the shopping experience, we found poor availability and wide inconsistencies in food labelling information in the e-grocery retail environment in Canada. Some variation in the availability and quality of key labelling information was observed between market-leading national brands and generic or private-label brands; however, the variability was greater across different websites than for brand types.

The poor availability and inconsistencies in key labelling information may hinder consumers from making informed purchasing decisions. Consistent with a previous study on the US e-grocery retail environment [16], a wide variability in the availability and quality of key labelling information was observed. With the lack of ability to physically assess products, e-grocery shoppers rely on the available product information to make informed purchasing decisions. However, the lack of information can inadvertently discourage purchasing certain foods, as seen with increased hesitancy in purchasing fresh produce online compared to brick-and-mortar stores [16]. It can also increase dependency on previous knowledge of products or limited information, resulting in increased brand-loyalty, price insensitivity, and favoring of processed and ultra-processed foods for their longer shelf-life [2,14,15]. Standardized labelling regulations for the e-commerce grocery retail environment are needed to ensure consistent, transparent, and legible information is available to Canadians, regardless of the retail place or environment of food purchasing.

The lack of information on websites may indirectly devalue the importance of the missing information in influencing purchasing decisions. Despite the high variability in the availability and quality of most labelling components, we found low availability of date marking, packaging, and recycling information throughout all websites. Date marking provides information on the perishability of foods to help consumers plan meals and reduce food waste, thereby playing a key role in the purchase decision-making process for many consumers [20,21]. With innovations in technology to improve the traceability of foods [22], retailers can easily present date marking information or, at a minimum, indicate shelf life (e.g., 6 weeks when refrigerated) to consumers. Similarly, packaging and recycling information can provide valuable information in helping consumers make sustainable purchasing decisions. Growing concerns with climate change have been driving innovations in sustainable food packaging; however, the environmental impact of food packaging is currently poorly communicated to and understood by the public [23,24]. Unrestricted by the physical boundaries of food labels, e-grocery retail platforms have the opportunity to highlight the significance of packaging materials on food quality and the environment, in order to narrow the current knowledge gap in consumers and create a more transparent and sustainable food system [23,24].

Simple and interpretive nutrition labelling, such as front-of-pack labelling, is important in helping consumers prioritize the nutritional characteristics of foods [4,25,26]. The poor accessibility of nutrition information (i.e., not presented as a part of the main text and in poor quality) may be related to very low use of nutrition information reported among online grocery shoppers (<4%) [27], compared with the relatively higher use of nutrition labels among traditional brick-and-mortar shoppers (>50%) [28]. Similar to traditional grocery shoppers, online grocery shoppers may rapidly compare multiple products using limited information available on packages or on websites [27]. Recognizing the complex food environment and consumers’ confusion when using nutrition information on the nutrition facts table, many governments are now introducing front-of-pack labels on pre-packaged foods [29,30,31]. The use of interpretive front-of-pack labelling systems can effectively communicate the nutritional quality of foods to help consumers make informed purchasing decisions, which will be equally important for e-grocery websites. For instance, e-grocery retail websites or mobile apps displaying simple front-of-pack labels have been shown to decrease the purchasing intentions of ‘less healthy’ foods [32,33], or purchases of nutrients of public health concerns (e.g., sugars [34], sodium [35,36]) compared with access to just NFts. National front-of-pack labelling systems, standardized for both on product packages and in the e-grocery retail environment, can be a key health promotion tool in helping consumers prioritize nutritional characteristics of foods in their purchasing decisions.

The e-grocery retail environment presents a unique opportunity to help consumers make healthy purchasing decisions with customization and targeted marketing [2,15,16]. Consistent with other studies [15], we found consumer-friendly features (e.g., re-purchase or ‘save’ foods), easy search functions for specific foods and beverages, and filter options based on dietary preferences (e.g., kosher, vegetarian, gluten free) or other factors (e.g., price, popularity) available to improve the shopping experience through customizations, using personal and other data collected from consumers. E-grocery retail platforms can also have the opportunity to promote healthy purchasing behaviors, by providing ‘healthier’ alternative product recommendations [37], exclusive online coupons or incentives [38,39], price discounts for ‘healthier’ foods [40], and/or nutrition messages targeted for consumers’ shopping behaviors. The use of accurate product descriptions and recommendations are also needed to ensure consumers are protected against misleading information, through an increased scope of the current food labelling regulations and/or establishment of new regulations specific for e-grocery retailers. Additionally, the clear and transparent communication of companies’ data sharing plans in all official languages of a country are needed to ensure consumers’ privacy and digital rights are protected.

Although the present study examined the availability and quality of food labelling components and other product information on the websites of the main grocery retailers in Canada, a few limitations should be noted. First, we did not take geographical variation into account when examining the accessibility of services. We set an urban geographical location, Toronto, ON, Canada; however, the service structure and fees of e-commerce grocery retailers may differ across Canada, particularly in rural communities. Further study is required to examine the potential gap in the availability of grocery services across Canada, to address potential inequity in the e-grocery retail environment. Second, we did not examine the differences in prices between brick-and-mortar stores and websites. Compared to customers experiencing the physical aspects of grocery shopping (i.e., selecting and purchasing foods), online shopping may increase labor or other overhead costs related to fulfilling and/or delivering orders for retailers. The increase in costs may be passed down to consumers through a user fee or an increase in the food cost. Additional research is needed to examine the financial impact on the accessibility of groceries when purchased online vs. brick-and-mortar stores. Third, we did not examine consumers’ perspectives on the e-grocery retail environment. Similar to physical packaging [4,20,28], e-grocery shoppers have been shown to use labelling information to make purchasing decisions [27]. However, considering the physical and technical differences of brick-and-mortar and e-grocery websites, further research is warranted to identify any specific labelling information and/or format needed to take into consideration to help e-grocery shoppers make informed purchasing decisions. Lastly, we did not examine the accuracy of product information. A recent study has shown that nutrition information on the UK grocery websites was almost identical to information available in brick-and-mortar stores [41]. In accordance with the *Foods and Drugs Act* [42], all available product information in Canada should not have any false, misleading, or deceptive information; however, human and technical errors may still result in some mismatch of product information available on websites. Standard labelling regulations can further help ensure accuracy in product information, as well as the consistency of label information presented in-store and online.

## 5. Conclusions

There was wide variability in the availability and quality of key labelling information in leading Canadian grocery retail websites. Despite many opportunities to enable a convenient grocery shopping experience, online grocery retail regulations are needed to ensure consistent product information is available to all consumers, regardless of the shopping method.

## Figures and Tables

**Table 1:** Summary Characteristics of the eight Canadian grocery websites ^1^.

Variables	*n* (%)
**Membership**	
Requires an account	8 (100)
Requires membership	2 (25)
Membership fee ^2^	2 (25)
**Pick up ^3^**	
Availability	5 (62.5)
Minimum purchasing amount	5 (62.5)
Service fee	3 (37.5)
**Delivery ^4^**	
Availability	7 (87.5)
Minimum purchasing amount	4 (50.0)
Service Fee	6 (75.0)
**General Website and Consumer-Friendly Features**	
Language ^5^	
English	8 (100)
French	5 (62.5)
Order history	7 (87.5)
Save lists or favorites	7 (87.5)
Substitution availability	6 (75.0)
Consumer feedback and/or ratings	2 (25.0)
**Privacy and Security Information**	
Accessibility of privacy statement	
Full, detailed statement in English	8 (100)
Statement in French	6 (75.0)
Statement in plain English	3 (37.5)
Collect “personally identifiable information”	8 (100)
Share data with a 3rd party	8 (100)
Data security explanation	6 (75.0)

^1^ Eight grocery websites (7 grocery retailers) were examined for accessibility, general function, and information on data privacy and security. Values are presented as total counts and proportion (%). ^2^ Membership fee ranged from $60–$120 a year. ^3^ Minimum purchasing amount for pick-up orders ranged from $30–$50, while service fee ranged from $2.97 to $5.00, depending on the date and time of the order pick up. ^4^ Minimum purchasing amount for delivery orders ranged from $35–$50, while service fee ranged from $3.00–$15.50, depending on the date, time, and location of the delivery. ^5^ English and French, two official languages in Canada, were assessed for availability. Detailed characteristics by website is shown in Table S2.

**Table 2:** Summary of the Availability and Quality of Labelling Information ^1^.

Variables ^2^	Mean (SD) or *n* (%)	*p*-Value for Brand Type ^5^	*p*-Value by Website ^6^
**Images**			
Total number of images available, mean (SD)	2.3 (1.9)	<0.001	0.7
Presence of front-of-pack image(s), *n* (%)	533 (96.0)	<0.001	<0.001
Presence of back-of-pack image(s), *n* (%)	69 (12.4)	0.01	<0.001
Presence of side image(s), *n* (%)	17 (3.1)	0.57	<0.001
Front-of-pack image quality			
(1) Can read everything without zooming in, *n* (%)	28 (5.3)	<0.001	0.005
(2) Can only read after zooming in, *n* (%)	95 (17.8)	0.12	<0.001
(3) Cannot read everything even after zooming in, *n* (%)	432 (81.1)	0.13	<0.001
**Nutrition information**			
**Comprehensive nutrition information ^3^ (*n* = 506), *n* (%)**	309 (61.1)	0.77	<0.001
**Nutrition Facts table ^3^ (*n* = 506)**			
Total available (as text and/or image), *n* (%)	348 (68.8)	0.23	<0.001
(1) Located on product information page, no scrolling required, *n* (%)	0		
(2) Located on product information page, but need to scroll to access, *n* (%)	123 (35.3)	0.38	<0.001
(3) At least one click away from the info page, *n* (%)	139 (39.9)	0.32	<0.001
(4) As product image only, *n* (%)	86 (24.7)	1.0	<0.001
**Ingredient information ^3^ (*n* = 506)**			
Total available (as text and/or image), *n* (%)	374 (73.9)	0.003	<0.001
(1) Located on product information page, no scrolling required, *n* (%)	1 (0.3)	1.0	0.54
(2) Located on product info page, but need to scroll to access, *n* (%)	85 (22.7)	0.87	<0.001
(3) At least one click away from the info page, *n* (%)	249 (66.6)	1.0	<0.001
(4) As product image only, *n* (%)	39 (10.4)	0.82	<0.001
**Allergen information ^3^ (*n* = 253)**			
Total available (as text and/or image), *n* (%)	136 (53.8)	0.11	0.003
(1) Located on product information page, with no scrolling required, *n* (%)	1 (0.7)	1.0	0.1
(2) Located on product information page, but need to scroll to access, *n* (%)	21 (15.6)	0.21	<0.001
(3) At least one click away from the info page, *n* (%)	88 (65.2)	0.06	<0.001
(4) As product image only, *n* (%)	26 (19.3)	0.46	<0.001
Nutrition and health claims ^5^			
As part of product description, *n* (%)	250 (45.0)	0.002	<0.001
Use of symbols developed by retailers, *n* (%)	69 (12.4)	0.03	<0.001
**Product recommendations**			
**Product recommendations by retailers ^4^, *n* (%)**	225 (40.5)	0.49	<0.001
**Price information**			
Per product, *n* (%)	555 (100)	1.0	1.0
Per reference amount (e.g., 100 g, 1 mL), *n* (%)	374 (67.4)	<0.001	<0.001
**Storage information**			
Storage information for all products, *n* (%)	121 (21.8)	<0.001	<0.001
For refrigerated products only (*n* = 235), *n* (%)	59 (25.1)	0.04	0.003
**Package and recycling information**			
**Package information (e.g., glass jar, plastic bottle), *n* (%)**	11 (2.0)	0.30	<0.001
**Recycling information, *n* (%)**	10 (1.8)	0.46	0.45

^1^ Out of 85 pre-selected products, not all products were available on all of the 8 websites. Therefore, a total of 555 products (413 brand-specific products from market leaders and 142 generic or private-label products) were examined on 8 grocery websites from seven Canadian grocery retailers. Date marking was not available for any of the products. ^2^ The availability of nutrition facts table (NFt), ingredient, and allergen information were examined only for products that needed to carry the nutrition information, as per the *Food and Drugs Regulations* [12], *n* = 506. ^3^ Product recommendations included advertisements of similar, complimentary, and/or other non-related sponsored products. ^4^ Nutrient content, health, and other food claims regulated by Health Canada Agriculture and Agri-Food Canada (e.g., “no added sugars”, “high source of fiber”, “organic”) were assessed for availability as part of the website product description (i.e., not part of the food images). ^5^ Student’s t-test and Fisher’s exact test were used to test for the difference between brand-specific and generic or private-label products. ^6^ Analysis of variance and chi-squared tests were used to test for the difference in the availability of information between websites. Statistical significance was set at *p* < 0.05.

## Data Availability

Full datasets can be obtained from the corresponding author at mary.labbe@utoronto.ca.

## Supplementary Materials

The following are available online at https://www.mdpi.com/article/10.3390/nu13082611/s1. Table S1: Characteristics of 85 Pre-Selected Products Searched; Table S2: Detailed Characteristics of the Eight Grocery Retail Websites; Table S3: Summary of the Availability and Quality of Key Labelling Information by Brand Type; Table S4: Summary of the Availability and Quality of Key Labelling Information by Website.
