# Supplementary material for: The Availability and Quality of Food Labelling Components in the Canadian E-Grocery Retail Environment
Source: Nutrients. 2021 Jul 29;13(8):2611. doi: 10.3390/nu13082611 (PMC8399674; doi:10.3390/nu13082611)
Supplement: Supplementary file 1 [file nutrients-13-02611-s001.zip › nutrients-1294000-supplementary.pdf]

Supplementary Tables

Table of Contents

Table S1. Characteristics of 85 Pre-Selected Products Searched.. 2

Table S2. Detailed Characteristics of the Eight Grocery Retail Websites 8

Table S3. Summary of the Availability and Quality of Key Labelling Information by Brand Type 10

Table S4. Summary of the Availability and Quality of Key Labelling Information by Website .. 12

Table S1. Characteristics of 85 Pre-Selected Products Searched.

Product Name	Brand	Product Size	Allergen Presence	Storage Condition	Packaging Material
White bread, sliced	Dempster	675 g	Yes	SS	BA
Freshly baked white dinner rolls*	None & Variety (Longo's)	NA	Yes	SS	BA
Plain bagel, gluten-free	Udi	397 g	Yes	FZ	BA
Marble pound cake*	Variety (Crispy Marble, Scaaf, Casablanca, Farmer's market)	300 - 500 g	Yes	SS	PL
Marble cake	McCain	510 g	Yes	FZ	RM
Coffee, classic roast	Folger's	920 g & 1.36 kg	No	SS	PL
Coffee mate powder, original	Nestle	450 g, 1.4 kg, 1.9 kg	Yes	SS	AL
Coke, original	Coca Cola	12 x 355 mL	No	SS	AL
Coke, Diet	Pepsi	2 L	No	SS	PL
Iced tea, original, powder mix	Nestea	715 g & 1 kg	No	SS	AL
Spaghetti, dry	Barilla	340 g bag	Yes	SS	CB
White rice, instant	Minute rice	700 g & 3 kg	No	SS	CB
Cheerios, original	General Mills	570 g & 1 kg	Yes	SS	CB
Froot loops	Kellogg's	345 g & 1.1 kg	Yes	SS	CB
Almond granola bars, sweet & salty	Nature Valley	210 g (6 bars) & 1.12 kg (32 bars)	Yes	SS	CB
Milk, 1% partly skim milk*	Variety (Sealtest, Beatrice, Natrel, President's Choice Organic)	4 L	No	RF	BA
Chocolate milk*	Variety (Sealtest, Beatrice, Natrel, Neilsen)	750 mL, 1 L, 2L	No	RF	CB

Yogurt, 100 g pack flavoured	Danone Activia	8 x 100 g & 24 x 100 g	No	RF	PL
Drinkable yogurt, flavoured	Yop	6 x 200 mL & 12 x 200 mL	No	RF	PL
Soy milk, unsweetened and enriched	Natura	PK	Yes	SS	RM
Cheese, cheddar	Cracker barrel	400 g bar	No	RF	BA
Ice cream, French vanilla	Breyers	1.66L	No	FZ	CB
Pudding, chocolate	Snack pack	396 g (4 cups)	Yes	SS	PL
Lolly water ice	Chapmans	28 bars	No	FZ	CB
Whipped frosting, vanilla	Betty Crocker	340 g	Yes	SS	AL
Pumpkin filling	ED Smith	540 mL	Yes	SS	AL
Eggs, fresh*	Variety (Goldegg, Burnabee, No Name)	NA	No	RF	CB
Egg whites*	Variety (Goldegg, Kirkland Signature, Natureegg)	500 g	Yes	RF	CB
Canola oil	Mazola	1.42 L	No	SS	PL
Mayonnaise	Hellmann's	445 mL, 890 mL, 1.8 mL	No	SS	PL
Butter, unsalted	Latantia	454 g & 2x1.22 kg	Yes	RF	BA
Margarine, light	Becel	454 g & 907 g	Yes	RF	PL
Salmon, fresh*†	None & Variety (Your Fresh Market)	NA	No	RF	BA
Shrimp, frozen*	Variety (Great Value, Longo's Kirkland Signature,	300- 500 g bag	No	FZ	BA

	Compliments, Irresitables, Seaquest)				
Chunk light tuna, skipjack in water	Clover leaf	170 g	No	SS	CB
Apple, royal gala*†	None	NA	No	SS	BA
Strawberries, whole, frozen*	Variety (Great Value, Kirkland Signature, Compliment's, President's Choice)	600 g & 2 kg	No	FZ	BA
Peaches, diced	Dole	4 x 107 mL & 20 x 107 mL	No	SS	PL
Orange juice, no pulp	Tropicana	1.54 L	No	RF	PL
Orange juice, frozen concentrate	Minute Maid	295 mL	No	FZ	CB
Tofu, medium firm	Sunrise	454 g	Yes	RF	PL
Red kidney beans, dry*	Variety (Unico, Siatra, Nupak)	750 g bag	No	SS	BA
Lentils, canned*	Variety (Unico, Primo, Great Value)	540 mL	No	SS	CB
Ground beef, extra lean*	None	NA	No	RF	BA
Chicken breasts, skinless, boneless*†	None	NA	No	RF	BA
Chicken breast nuggets, fully cooked, pub style	Jane's	700 g	Yes	FZ	CB
Plant-based burgers	Beyond Meat	227 g	No	RF	BA
Black forest ham	Maple leaf	175 g	No	RF	PL
Basil, dry†	Club house	37 g	No	SS	PL
Montreal steak spice seasoning†	Club house	60 g	No	SS	PL

Cake mix, chocolate fudge, super moist cake	Betty Crocker	432 g	Yes	SS	CB
Macaroni & cheese pack, original	Kraft Dinner	225 g	Yes	SS	CB
Sandwich with deli-meat, single serve, store made*†	None & Variety (Longo's)	Variety	M	RF	BA
Salisbury steak meal, frozen	Hungry Man	455 g	Yes	FZ	CB
Vegetarian lasagna, frozen*	Variety (Our Finest, Stouffer's, Kirklan signature)	291 g	Yes	FZ	CB
Pizza, pepperoni, rising crust	Delissio	788 g	Yes	FZ	CB
Peanut butter, smooth	Kraft	500 g & 2 kg	Yes	SS	PL
Almond butter, smooth	Nuts to you	227 g	Yes	SS	GL
Almonds, sliced*	Variety (Great Value, Compliment's, Selection, No Name)	100-200 g	Maybe‡	SS	BA
Potatoes, russet*†	None	5 lb bag	No	SS	BA
Super fries straight cut	McCain	650 g bag	No	FZ	BA
Tuna snack, spicy Thai chili	Clover leaf	90 g	Yes	SS	CB
Greek salad pack*	None & Variety (Fresh Attitude)	single serving size	Maybe‡	RF	NA
Kale salad kit	Eat smart	340 g	Yes	RF	BA
Hummus, classic*	Variety (Fontaine Sante, Summer Fresh, President's Choice)	454 g & 2 x 565 g	Yes	RF	PL
Ketchup	Heinz	1 L & 2x1.25 L	No	SS	PL
Teriyaki sauce	VH	341 mL	Yes	SS	GL

Pasta sauce, Hunt's Thick & Rich	Hunt's	680 mL	No	SS	AL
Potato chips, original	Pringle's	148 g	Yes	SS	AL
Oreo's, original	Christie's	303 g	Yes	SS	PL
Goldfish crackers, cheddar	Pepperidge Farm	200 G	Yes	SS	CB
Chicken noodle soup, canned, home style	Campbell	284 mL	Yes	SS	AL
Chicken noodle dry soup mix	Lipton	338 g	Yes	SS	CB
Vegetable bouillon cubes	Knorr's	69 g	No	SS	CB
Swiss milk chocolate bar	Lindt	100 g	Yes	SS	BA
Jam, strawberry	Smucker's	500 mL	No	SS	GL
Chocolate hazelnut spread	Nutella	725 g	Yes	SS	PL
Licorice, strawberry	Twizzlers	227 g	No	SS	BA
Lettuce, romaine*†	None	NA	No	RF	BA
Peas, frozen	Green Giant	750 g	No	FZ	BA
Dill pickles, garlic	Bick's	1 L	No	RF	GL
Diced tomatoes, canned, no salt added	Unico	796 mL	No	SS	AL
Baby cereal, rice & banana	Gerber	227 g	Yes	SS	CB
Pureed peas (for > 6 months-old)	Heinz	128 mL	No	SS	GL
Pureed baby food in pouch*	Variety (Baby Gourmet, Gerber Organic, Parent's Choice Organic)	128 mL	Maybe‡	SS	BA

*Indicates products with no specific brands or with variety of brands were treated as generic- or private-labelled products. †Indicates products not required to carry a mandatory Nutrition Facts table in Canada. ‡Indicates products that may have allergens based on the ingredients. Abbreviations: AL, aluminum; BA, plastic bag or wrap; CB, cardboard; FR, frozen; GL, glass; PL, plastic; RF, refrigerated; RM, mixed recyclable material; SS, shelf-stable (i.e., room temperature).

Table S2. Detailed Characteristics of the Eight Grocery Retail Websites[1]

	Costco	Costco & Instacart	Grocery Gateway	Loblaws	Metro	No Frills	Voilà	Walmart	n (%)
Membership									
Require an account	✓	✓	✓	✓	✓	✓	✓	✓	8 (100)
Require membership	✓	✓							2 (25)
Membership fee	✓ ($60-120)	✓ ($60-120)							2 (25)
Pick-up									
Availability				✓	✓	✓	✓	✓	5 (62.5)
Minimum purchasing amount				✓ ($30)	✓ ($50)	✓ ($30)	✓ ($35)	✓ ($35)	5 (62.5)
Service fee				✓ ($3-$5)		✓ ($3-$5)		✓ ($2.97-$4.97)	3 (37.5)
Delivery									
Availability	✓	✓	✓	✓	✓		✓	✓	7 (87.5)
Minimum purchasing amount		✓ ($35)			✓ ($50)		✓ ($50)	✓ ($35)	4 (50.0)
Service fee	✓ ($3)		✓ ($7.99-$15.50)	✓ ($9.95)	✓ ($11.99)		✓ ($7.99)	✓ ($7.99-$12.97)	6 (75.0)
Other consumer-oriented and/or easy access features									
Language									
English	✓	✓	✓	✓	✓	✓	✓	✓	8 (100)
French	✓	✓		✓	✓			✓	5 (62.5)
Order history	✓	✓	✓	✓	✓	✓		✓	7 (87.5)
Save lists or favorites		✓	✓	✓	✓	✓	✓	✓	7 (87.5)
Substitution availability		✓	✓	✓	✓	✓		✓	6 (75.0)
Consumer feedback/ratings			✓					✓	2 (25.0)

Privacy & Security information									
Accessibility of privacy statement									
Full, detailed statement in English	✓	✓	✓	✓	✓	✓	✓	✓	8 (100)
Statement in French	✓	✓	✓	✓	✓	✓			6 (75.0)
Statement in plain English				✓	✓	✓			3 (37.5)
Collect "personally identifiable information"	✓	✓	✓	✓	✓	✓	✓	✓	8 (100)
Share data with a 3rd party	✓	✓	✓	✓	✓	✓	✓	✓	8 (100)
Data security explanation	✓	✓	✓	✓		✓		✓	6 (75.0)

[1]A total of 8 grocery websites from 7 Canadian grocery retailers were examined and included in the study. ✓indicates websites with examined characteristics.

Table S3. Summary of the Availability and Quality of Key Labelling Information by Brand Type

**Variables[1,2]**	**Brand-specific (n=413)**	**Generic or private-label (n=142)**	***p*-value[6]**
Images			
Total number of images available, mean (SD)	2.5 (2.1)	1.6 (1.0)	<0.001
Presence of front-of-pack image(s), n (%)	411 (99.5)	122 (85.9)	<0.001
Presence of back-of-pack image(s), n (%)	60 (14.5)	9 (6.3)	0.01
Presence of side panel image(s), n (%)	14 (3.4)	3 (2.1)	0.57
Front-of-pack image quality, n (%)			
A) Can read everything without zooming in, n (%)	8 (1.9)	20 (14.1)	<0.001
B) Can only read after zooming in, n (%)	77 (18.6)	18 (12.7)	0.12
C) Cannot read everything even after zooming in, n (%)	328 (79.4)	104 (73.2)	0.13
Nutrition information [3]			
Comprehensive nutrition information, n (%)	228 (56.0)	81 (81.8)	0.77
Nutrition Facts table			
Total available (as text and/or image)	285 (70.0)	63 (63.6)	0.23
1) Located on product information page, with no scrolling required, n (%)	0	0	N/A
2) Located on product information page, but need to scroll to access, n (%)	104 (25.6)	19 (29.3)	0.38
3) At least one click away from the info page, n (%)	110 (27.0)	29 (15.2)	0.32
4) As product image only, n (%)	71 (17.4)	15 (15.2)	1.0
Ingredient information			
Total available (as text and/or image), n (%)	313 (76.9)	61 (61.6)	0.003
1) Located on product information page, no scrolling required, n (%)	1 (0.2)	0	1.0
2) Located on product info page, but need to scroll to access, n (%)	72 (17.7)	13 (13.1)	0.87
3) At least one click away from the info page, n (%)	208 (51.1)	41 (41.4)	1.0
4) As product image only, n (%)	32 (7.9)	7 (7.1)	0.82
Allergen information			
Total available (as text and/or image), n (%)	124 (57.9)	12 (41.4)	0.11
1) Located on product information page, with no scrolling required, n (%)	1 (0.5)	0	1.0
2) Located on product information page, but need to scroll to access, n (%)	21 (9.8)	0	0.21
3) At least one click away from the info page, n (%)	77 (36.0)	11 (37.9)	0.06
4) As product image only, n (%)	25 (11.7)	1 (3.4)	0.46
Product recommendations [4]			
Product recommendations by retailers	171 (41.4)	54 (38.0)	0.49
Nutrition and health claims [5]			
As part of product description, n (%)	204 (49.4)	46 (32.4)	0.002
Use of symbols developed by retailers, n (%)	48 (11.6)	21 (14.8)	0.03
Price information			
Per product, n (%)	413 (100)	142 (100)	1.0

Per reference amount (e.g., 100 g, 1 mL, each), n (%)	304 (73.6)	70 (49.3)	<0.001
Storage information			
Storage information for all products, n (%)	78 (18.9)	43 (30.3)	<0.001
For refrigerated products only, n (%)	37 (30.3)	22 (15.5)	0.04
Package and recycling information			
Package information (e.g., glass jar, plastic bottle), n (%)	10 (2.4)	1 (0.7)	0.30
Recycling information, n (%)	9 (2.2)	1 (0.7)	0.46

[1] Out of 85 pre-selected products, not all products were available on all the selected websites. A total of 555 products (413 brand-specific products from category market leaders and 142 generic or private-label products) were available and searched on the 8 grocery websites from seven Canadian grocery retailers.

[2] Date marking was not available for any of the products.

[3] The availability of nutrition facts table, ingredient, and allergen information were examined only for products that were mandated to carry the nutrition information in Canada.

[4] Nutrient content, health, and other food claims regulated by Health Canada or Agriculture and Agri-Food Canada (e.g., "no added sugars", "high source of fiber", "organic") were assessed for availability as part of the website product description (i.e., not part of the food images).

[5] Product recommendations made by retailers were assessed, including "similar products," "alternatives," or "sponsored."

[6] Student's t-test and Fisher's exact test were used to test for the difference between brand-specific and generic or private-label products. Statistical significance was set at $p<0.05$.

Table S4. Summary of the Availability and Quality of Key Labelling Information by Website

Variables [1,2]	Costco	Costco & Instacart	Grocery Gateway	Loblaws	Metro	No Frills	Voilà	Walmart	p-value [6]
Number of assessed products	16	27	61	63	62	61	62	61	
Images									
Total number of images available, mean (SD)	2.5 (1.0)	1.0 (0)	1.0 (0)	2.4 (1.5)	1.3 (0.9)	2.4 (1.5)	4.1 (2.2)	4.7 (2.7)	<0.001
Presence of front-of-pack image(s), n (%)	16 (100)	27 (100)	61 (100)	62 (98.4)	61 (98.4)	61 (100)	62 (100)	61 (100)	0.70
Presence of back-of-pack image(s), n (%)	0	0	0	0	1 (1.6)	0	9 (14.5)	4 (6.6)	<0.001
Presence of side panel image(s), n (%)	0	0	0	0	1 (1.6)	0	10 (16.1)	6 (9.8)	<0.001
Front-of-pack image quality									
1) Can read everything without zooming in, n (%)	0	0	0	5 (7.9)	0	3 (4.9)	0	0	0.005
2) Can only read after zooming in, n (%)	14 (87.5)	0	0	5 (7.9)	0	3 (4.9)	0	55 (90.2)	<0.001
3) Cannot read everything even after zooming in, n (%)	2 (12.5)	27 (100)	61 (100)	53 (84.1)	62 (100)	55 (90.2)	62 (100)	6 (9.8)	<0.001
Nutrition information [3]									
Comprehensive nutrition information, n (%)	9 (56.2)	0	40 (66.7)	47 (75.8)	5 (8.2)	45 (75.0)	38 (62.3)	44 (73.3)	<0.001
Nutrition Facts table, n (%)									
Total available (as text and/or image), n (%)	14 (87.5)	0	51 (85.0)	56 (90.3)	4 (6.6)	54 (90.0)	57 (93.4)	49 (81.7)	<0.001
1) Located on product information page, with no scrolling required, n (%)	0	0	0	0	0	0	0	0	N/A
2) Located on product information page, but need to scroll to access, n (%)	0	0	51 (100)	0	0	0	53 (93.0)	0	<0.001
3) At least one click away from the info page, n (%)	0	0	0	56 (100)	0	54 (100)	0	0	<0.001
4) As product image only, n (%)	14 (100)	0	0	0	4 (100)	0	4 (7.0)	49 (100)	<0.001
Ingredient information									
Total available (as text and/or image), n (%)	11 (68.7)	0	52 (86.7)	56 (90.3)	38 (62.3)	54 (90.0)	42 (68.8)	60 (100)	<0.001

1) Located on product information page, no scrolling required, n (%)	0	0	1 (1.9)	0	0	0	0	0	0.54
2) Located on product info page, but need to scroll to access, n (%)	0	0	51 (98.1)	0	0	0	21 (50.0)	0	<0.001
3) At least one click away from the info page, n (%)	0	0	0	56 (100)	38 (100)	54 (100)	0	60 (100)	<0.001
4) As product image only, n (%)	11 (100)	0	0	0	0	0	21 (50.0)	0	<0.001
Allergen information									
Total available (as text and/or image), n (%)	5 (55.6)	0	15 (48.4)	22 (66.7)	9 (28.1)	20 (64.5)	20 (62.5)	22 (68.7)	0.003
1) Located on product information page, with no scrolling required, n (%)	0	0	1 (6.7)	0	0	0	0	0	0.1
2) Located on product information page, but need to scroll to access, n (%)	0	0	14 (93.3)	0	0	0	5 (25.0)	0	<0.001
3) At least one click away from the info page, n (%)	0	0	0	22 (100)	7 (77.8)	20 (100)	0	22 (100)	<0.001
4) As product image only, n (%)	5 (100)	0	0	0	2 (22.2)	0	15 (75.0)	0	<0.001
Nutrition and health claims [4]									
As part of product description, n (%)	13 (81.2)	19 (70.4)	13 (21.3)	28 (44.4)	33 (53.2)	26 (42.6)	13 (21.0)	59 (96.7)	<0.001
Use of symbols developed by retailers, n (%)	0	0	9 (14.8)	0	32 (51.6)	0	7 (11.3)	0	<0.001
Product recommendations [5]									
Product recommendations by retailers, n (%)	16 (100)	27 (100)	0	58 (92.1)	8 (12.9)	6 (9.8)	0	56 (91.8)	<0.001
Price information									
Per product, n (%)	16 (100)	27 (100)	61 (100)	63 (100)	62 (100)	61 (100)	62 (100)	61 (100)	1.0
Per reference amount (e.g., 100 g, 1 mL, each), n (%)		0	0	62 (98.4)	62 (100)	61 (100)	61 (98.4)	58 (95.1)	<0.001
Storage information			0						
Storage information for all products, n (%)	0	7 (25.9)	3 (4.9)	2 (3.2)	2 (3.2)	1 (1.6)	5 (8.1)	58 (95.1)	<0.001
For refrigerated products only (n=235), n (%)	0	1 (25.0)	1 (9.1)	0	0	0	0	2 (100)	0.003
Package and recycling information									
Package information (e.g., glass jar, plastic bottle), n (%)	3 (18.7)	1 (3.7)	0	0	0	0	5 (8.1)	1 (1.6)	<0.001

Recycling information, n (%)	0	0	0	1 (1.6)	0	1 (1.6)	2 (3.2)	0	0.45

[1] Sixty-three unique brand-specific products from category market leaders were searched on eight grocery websites from seven Canadian grocery retailers. Since not all products were available on all the selected websites, a total of 413 product were searched.

[2] Date marking was not available for any of the products.

[3] The availability of nutrition facts table, ingredient, and allergen information were examined only for products that were mandated to carry the nutrition information in Canada (n=407).

[4] Nutrient content, health, and other food claims regulated by Health Canada or Agriculture and Agri-Food Canada (e.g., "no added sugars", "high source of fiber", "organic") were assessed for availability as part of the website product description (i.e., not part of the food images).

[5] Product recommendations made by retailers were assessed, including "similar products," "alternatives," or "sponsored."

[6] Analysis of variance and chi-square tests were used to assess the difference in the availability of information across websites. Statistical significance was set at $p<0.05$.
